# Investigating the temporal relationship between individual-level social capital and health in fragile families

**DOI:** 10.1186/s12889-015-2437-3

**Published:** 2015-11-16

**Authors:** Kim Nichols Dauner, Neil A. Wilmot, Jennifer F. Schultz

**Affiliations:** Health Care Management Program / Department of Economics, University of Minnesota Duluth, 1318 Kirby Drive, Duluth, MN 55812 U.S.A.; Department of Economics, University of Minnesota Duluth, 1318 Kirby Drive, Duluth, MN 55812 U.S.A.

**Keywords:** Health, Individual social capital, Fragile families, Logistic regression

## Abstract

**Background:**

The potential for social capital to influence health outcomes has received significant attention, yet few studies have assessed the temporal ordering between the two. Even less attention has been paid to more vulnerable populations, such as low-income women with children. Our objective was to explore how different dimensions of social capital impact future health status among this population.

**Methods:**

This study uses data from the Fragile Families and Child Well-Being (FFCWB) Study, which has followed a cohort of children and their families born in large U.S. cities between 1998 and 2000 to mostly minority, unmarried parents who tend to be at greater risk for falling into poverty. Four separate measures of social capital were constructed, which include measures of social support and trust, social participation, perceptions of neighborhood social cohesion, and perceptions of neighborhood social control. The temporal effect of social capital on self-reported health (SRH) is investigated using logistic regression and we hypothesize that higher levels of social capital are associated with higher levels of self-rated health.

**Results:**

After controlling for socioeconomic and demographic factors related to social capital and self-rated health, social support and trust, perceptions of neighborhood social cohesion and control at an earlier point in time were positively associated with higher levels of health four-years later. Social participation was not related to increased health. The empirical results appear robust.

**Conclusion:**

Higher levels of social capital are predictive of improved health over a four-year time frame. These results suggest that policy initiatives supporting increasing the social capital available and accessible by low-income, urban, minority women are viable for improving health. Such policies may have the potential to reduce socioeconomic health disparities.

## Background

There has been much research on the relationship between social capital and health. However, much of the research to date has been cross-sectional in nature and at the country level. Given that within a country population subgroups may experience social capital in ways that are different from that of the dominant group, this study focuses on the relationship between social capital and self-rated health (SRH) in a sample of urban, low-income mothers that are predominately racial and ethnic minorities. Moreover, we examine the relationship temporally, which is an advantage over cross-sectional social capital-SRH studies.

Originally framed at the societal level, social capital as defined by Putnam, refers to a combination of social organizations, social networks, and civic participation that can improve the efficiency of society by facilitating coordinated action [1]. This definition characterizes social capital as a form of social cohesion generated at the societal level. It is also recognized that social capital can be generated at the individual level through one’s ability to access the benefits of social networks and structures [2]. In public health, social capital is viewed as a multidimensional construct including individual and community level dimensions that acts as a determinant of health [3, 4].

There is considerable evidence of a relationship between individual and community level social capital and good health, with trust, social participation, and reciprocity being the dimensions of social capital appearing to have the strongest relationship with health [5–7]. At the individual level social capital involves not only the ability to access social networks and resources but assumes the ability to leverage and influence social relationships [8]. Important in this conceptualization is the assumption of reciprocity between individual and community. In other words individuals both use and generate social capital. Snelgrove and colleagues frame individual perceptions of social capital as a collective benefit derived from generalized trust and community engagement rather than solely an individually experienced resource [9]. Similarly, Poortinga found evidence of interactions between social capital at the national level and social trust and civic participation at the individual level, suggesting individual willingness to engage with others influences the relationship between community and individual levels of social capital [10]. This has important ramifications for measurement. On it’s own social capital is not directly observable and there are limitations to aggregate measures of it; thus it’s presence is inferred from the measurement of observable antecedents such as trust and participation [11–13]. In addition to these cognitive pathways between social capital and health, research suggests that household, neighborhood, and community characteristics contribute to the social capital-health relationship [14].

The cross-sectional nature of many studies has made temporal ordering impossible; however, more recent literature suggests that certain dimensions of social capital at earlier time points are positively associated with self-rated health at later points in time. Examining changes in health status over time, Giordano and Lindström, found a significant association with inability to trust and deteriorating health status [15]. They also found a similar association between generalized trust of others and better psychological health over time [16]. Another study that used data from the British Household Panel Survey found that generalized trust and social participation at an earlier time point predicted self-rated health at a later time point, after adjusting for other health determinants [17]. This temporal relationship between trust and social capital remained even after accounting for household context [18]. Others have found a positive, temporal relationship between individual community service group membership and neighborhood trust and SRH over a two-year time period [19]. In addition, Lamarca and colleagues found that higher levels of social capital were related to maintaining good health throughout pregnancy and the first six months post-partum [20]. This research also revealed that individual social capital explained more variation in health than community level factors. Two recent meta-analyses of prospective studies of the relationship between social capital and mortality demonstrate a mixed relationship between social capital and health. Nyqvist and colleagues found strong evidence for an inverse association between social participation and mortality and they found that this relationship persists across male and female genders [21]. They found weak evidence of a negative relationship between social networks and mortality. Choi and colleagues found little evidence of a relationship between seven dimensions of social capital and all-cause mortality, cardiovascular disease and cancer; however, they found a slight positive association between social and civic participation and mortality [22]. A lack of consistent measures of social capital weakened their ability to detect associations.

A criticism of the social capital literature is the assumption that social capital is gender and power blind and there are calls to further study the relationship between social capital and health according to race/ethnicity, gender, and socioeconomic status [7, 8, 11]. Particularly relevant to our work is the study of social capital among women, and in particular low-income women. Our study uses data from low-income, urban mothers participating in the Fragile Families and Child Wellbeing Study (FFCWB). This population is considered “fragile”, or vulnerable, because mothers who are not married to a child’s father at the time of that child’s birth have a greater risk of separating and living in poverty (for more on the economic hardships related to fragile families see Kalil & Ryan’s research) [23].

Research indicates that social capital varies by gender and income. Generally speaking, women report both more use of and provision of support [24]. This support, mainly conceptualized as social support, appears to protect health [25, 26]. In low-income communities, social capital may act as a buffer against health disparities related to socioeconomic inequality, and may even promote social mobility [27, 28]. At the same time, economic capital may be needed to generate and accumulate social capital for the benefit of health, thereby creating a dependency between the two [28]. Similarly, persons who are unsure of their ability to reciprocate support may avoid accessing support [29]. In a systematic review of 60 studies on the relationship between social capital and socioeconomic inequalities in health, Uphoff and colleagues, found evidence for both a buffer effect and a dependency effect of social capital on socioeconomic inequalities in health [28]. However, among persons of very low socioeconomic status, there is some evidence that social capital has a stronger buffer effect on health. In recognition of numerous limitations of the studies reviewed (e.g. few testing for the interaction between SES and social capital, mostly cross-sectional studies), the study authors suggest further study of the role of social capital in health inequalities.

The current study addresses several of these gaps by using data from a longitudinal cohort study of primarily urban, low-income mothers who are racial and ethnic minorities. We sought to identify a temporal association between social capital at an earlier time (referred to as [t-1]) and health outcomes at a later time (denoted as [t]). We used measures that are similar to those that have been employed in other studies, including social support and trust, social participation, and neighborhood collective efficacy, thus adding to the construct validity of such measures. We aim to assess social capital in a population more likely to be living in poverty, thereby contributing to the discussion of health in underserved populations. In addition, the population surveyed in the FFCWB study consists of mostly racial and ethic minorities, who have experienced persistent health disparities [30].

## Methods

### Data

Our study is a secondary analysis of data collected by the FFCWB study. We used publicly available data and registered for its use through Princeton University’s Office of Population Research. The FFCWB study follows a cohort of about 5,000 children born to low-income parents in 20 major U.S. cities (in 15 states), during the years 1998–2000 and who have been surveyed at regular intervals since birth. By design approximately three-quarters of the mothers were unmarried. Face-to-face interviews were conducted with 4898 mothers shortly after giving birth. Both parents were interviewed at the time of their infant’s birth and then again one, three, five, and nine years later. The study was designed to provide information on the conditions and capabilities of new parents, the determinants and trajectories of parental relationships, and the consequences for parents and their children of health, child welfare, and social service policies as well as other aspects of their environment [31].

We use data from the five and nine-year post-baseline follow-up interviews to assess the relationship between social capital and health outcomes. It is important to note that the questions related to social capital were not consistently measured across all waves of the FFCWB study which limits our ability to assess changes in social capital over time in relationship to health. We use data from only the mothers with regard to social capital and health, as there was a disproportionate response rate for the fathers beyond the initial wave of the FFCWB study.

### Measures

#### Dependent variable - self-rated health

We measured our dependent variable, SRH, using the data obtained during the nine-year wave of the survey. Mothers were asked to rate their overall general health on a five-point scale ranging from excellent to poor. Self-reported health has been shown to be a valid measure of general health and its use is consistent across studies examining the relationship between social capital and health. The five-point scale was recoded into a dichotomous variable ‘favorable’ (excellent, very good, good) and ‘unfavorable’ (fair, poor) health.

#### Measures of social capital

Based on individual’s responses to questions from the five-year follow up survey of mothers, four indices of social capital were created. The inclusion of questions within each index was informed by the 2006 Social Capital Community Survey [32]. Each index is described below.

#### Social support and trust

Mothers were asked about whether they had others in their life to provide them with emotional and tangible supports when needed. Specifically, mothers were asked whether they had someone that 1) they could trust to look after their child if they were away, 2) would loan them $200, 3) could provide them a place to live 4) could provide them with emergency child care, 5) co-sign for a bank loan ($1,000) and 6) they could share confidence with. Response options for each question were dichotomous (yes/no). The constructed measure of social support and trust is a summation of the “yes” (=1) responses to each question.

#### Social participation

This measure counts the number of “yes” responses by mothers to questions related to participation in various community entities. The three questions included participation in activities at the child’s school, community groups, and religious services. For the question on religious services, responses of once a week or more were characterized as participation for this index. A response of yes to any question indicated involvement or participation in the entity during the 12 months prior to completion of the survey.

#### Perceptions of neighborhood social cohesion

The FFCWB uses a validated measure of social cohesion [33]. The five questions comprising this index ask for the respondent’s level of agreement with the following statements: 1) willingness to help a fellow neighbor, 2) if neighborhood was viewed as a close-knit community, 3) if people generally get along with each other, 4) if they share the same values and, 5) if gangs were a problem in the neighborhood. The question regarding gangs being a problem was recoded so that lower levels of agreement were coded as being more positive. After recoding, the mean response to the Likert-type questions expressing level of agreement was used to construct the index, with higher scores indicating higher levels of neighborhood social cohesion.

#### Perceived neighborhood social control

The measure of perceived neighborhood social control also uses a validated measure within the collective efficacy construct and is constructed from five questions [33]. Using a Likert-type scale, mothers were asked about the likelihood of neighbors to intervene if children are 1) skipping school, 2) spray painting a building and, 3) showing disrespect to an adult. Additionally, the likelihood of a neighbor intervening to diffuse a fight, as well as a neighbor’s willingness to intervene to save a local firehouse were assessed and utilized. The index was constructed as a mean, based on an individual’s response to the above questions, with higher scores indicating higher levels of perceived neighborhood social control. While the four constructed measures of social capital are related, the pairwise correlation values are muted; the highest degree of correlation (0.48) is found between Perceived Neighborhood Social Cohesion and Perceived Neighborhood Social Control, while the remaining correlations are below 0.24.

#### Explanatory variables

Several socioeconomic, demographic, and behavioral variables are known to influence self-rated health. Such variables, which were controlled for, included level of education, age, race, income, relationship status, employment status, number of children, poverty, smoking behavior, and SRH at (t-1). Education was measured as highest educational level obtained. Age was categorized into four categories <25 years, 25–34 years, 35–44 years and 45 years and above. Race is categorized by the FFCWB as “black” “Hispanic” “white” or “other.” Income was categorized into three categories of less than $30,000, $30,000–$59,999, and $60,000 and above. Since income was missing a fourth category “missing income” was included. Relationship status was measured as two separate binary variables as to married or not married, cohabitating or not cohabitating to get at the diversity of relationship types among women in the sample. Employment status was a binary measure “yes” or “no.” The number of children the woman has was a continuous measure. Poverty status was a binary measure equaling one if the individual has received welfare or food stamps in the previous 12 months. Whether the person has smoked within the last 30 days was a binary “yes” or “no” measure. SRH at (t-1) was categorized the same way the dependent variable was categorized. All measures, with the exception of education which comes from the baseline interview, come from the five-year follow-up interview (t-1).

#### Analysis

Our empirical model is adapted from Bolin et al.’s theoretical model with the family as producer of social capital and health, and with the amount of social capital being positively related to level of health [34]. We account for the temporal ordering using measures of social capital from an earlier time point in reference to health measures; interest lies in examining the temporal relationship over a four-year period between the explanatory variables, lagged one period (t-1), and the current period for self-reported health. In particular, we tested the hypothesis that social capital at time (t-1) is positively associated with SRH at time (t). To examine this hypothesis a logistic regression model is estimated, including socio-economic and demographic variables. The equation for the logistic model is given as:

1 $$ \mathrm{Logit}\left({H}_t\right)=\beta {X}_{t-1}+\varphi {S}_{t-1}+{\varepsilon}_t $$

where *H*_*t*_ is *SRH* in period *t*, *X*_*t-1*_ includes socio-economic and demographic variables in period *t-1*, and *S*_*t-1*_ represents the individual measure of social capital, in period *t −1*. The hypothesis is represented by *φ*, which is expected to be positive. Each dimension of social capital was included individually in a model examining its impact on SRH, while controlling for the covariates (models 1–4). A fifth model which includes all measures of social capital was examined. In a similar vein, ordered logistic regression models were estimated as a robustness check. Marginal effects were calculated for each social capital construct on SRH.

## Results

### Study sample

Of the original 4898 mothers participating in the initial Fragile Families interview, 4139 (84.5 %) participated in the five-year interview, and 3512 (71.7 %) participated in the nine-year interview. While the dependent variable, SRH, is obtained from the nine-year interview, covariates were constructed from the five-year interview. This restricts the sample to women who participated in both the five and nine-year waves of the study and results in a sample of 3,284 women. It should be noted that the sample sizes of several models have fewer observations as a result of non-response to interview questions.

Frequencies for the individual social capital measures and explanatory variables by self-reported health are presented in Table 1. The majority of the sample is considered in favorable health, with approximately 16 % reporting unfavorable health. Of those who reported annual income, 48.5 % are earning less than $30,000 per year, the median reported income of the sample. Approximately 40 % have received income from welfare/TANF or from food stamps, in the last 12 months (*poverty*). Additionally, nearly half the sample is black, and two-thirds are nonsmokers. To examine the potential role of selection bias, we compared individuals who failed to respond to the nine-year interview with those individuals who remained, no statistical differences were observed in SRH, three of the four social capital measures, income, and age variables. Only the constructed social participation measure was found to be lower for the group of mothers who did not follow through, yet the logistic results suggest the social participation measure itself is not statistically significant in explaining health. As such, there is little evidence to suggest that selection bias is important.

**Table 1:** Frequency of variables stratified by self reported health

			Health Status			
			Favorable		Unfavorable	
	Frequency	Mean	Frequency	Mean	Frequency	Mean
Total	3284		86.1 %		13.9 %	
Indicies of Social Capital						
Social Support and Trust	100.0 %	4.79	86.1 %	4.88	13.9 %	4.19
Social Participation	100.0 %	1.12	86.1 %	1.13	13.9 %	1.05
Percevied Neighborhood						
Social Control	98.6 %	3.24	85.0 %	3.26	13.6 %	3.13
Percevied Neighborhood						
Social Cohesion	99.3 %	2.99	85.7 %	3.02	13.6 %	2.79
Explanatory Variables						
Health _*t*-*1*_	100.0 %		86.1 %		13.9 %	
Married	35.4 %		31.6 %		3.8 %	
Cohabitating	60.3 %		52.6 %		7.7 %	
Race						
White	21.7 %		19.0 %		2.7 %	
Black	50.4 %		43.3 %		7.1 %	
Hispanic	24.3 %		20.7 %		3.6 %	
Other	3.4 %		3.1 %		0.3 %	
Poverty	42.1 %		33.9 %		8.3 %	
Income						
Less than $30,000	36.7 %		29.9 %		6.9 %	
$30,000–$59,999	24.7 %		22.2 %		2.5 %	
$60,000 +	14.2 %		13.5 %		0.7 %	
Missing Income	24.4 %		20.6 %		3.8 %	
Age						
Less than 25	17.0 %		15.1 %		1.9 %	
25–34	60.0 %		51.2 %		8.8 %	
35–44	20.8 %		18.1 %		2.7 %	
45+	2.2 %		1.7 %		0.5 %	
Employed	60.1 %		53.5 %		13.9 %	
Education						
Less than High School	37.3 %		30.9 %		6.4 %	
High School Graduate	26.3 %		22.7 %		3.5 %	
Some College	25.2 %		21.8 %		3.4 %	
College Graduate	11.1 %		10.7 %		0.4 %	
Nonsmoker	70.1 %		62.1 %		8.0 %	
Number of Children						
1	18.8 %		16.9 %		1.9 %	
2	35.2 %		30.7 %		4.4 %	
3	23.5 %		20.1 %		3.4 %	
4 or More	22.4 %		18.3 %		4.1 %	

Favorable health is considered a Self-Reported health value of 5 (Excellent; *n* = 736), 4 (Very Good; 1119) or 3 (Good; 1073), while Unfavorable is comprised of a SRH outcome of 2 (Fair; 510) and 1 (Poor; 74). Poverty is defined as having received welfare or foodstamps within the last 12 months. The mean is provide for the measures of social capital

The estimated odds ratios of the multiple logistic regressions investigating the relationship between SRH and the measures of social capital are presented in Table 2, along with the robust standard errors. The results indicate that, with the exception of social participation (Table 2, Model 2), the measures of social capital are statistically significant and have the expected positive signs. The results suggest that women with higher levels of social support and trust, who report increased social control and cohesion within their neighborhoods (t-1), rate themselves to be healthier at time (t). The results for social support and trust indicate that a unit increase in the social support measure leads to an increase in the odds of reporting good health by approximately 11 %. The marginal effects, presented in Table 3, suggests that a unit increase in an individual’s level of social support increases the probability of reporting favorable health status by nearly 1 %. The increases in the probability of reporting favorable health status observed for the social capital measures of perceived neighborhood social control and perceived neighborhood social cohesion are much higher, 1.7 % and 2.9 %, respectively.

**Table 2:** Odds ratios for social capital measures on self reported health

	Model 1		Model 2		Model 3		Model 4		Model 5	
*n*	3284		3284		3237		3261		3231	
Indicies of Social Capital										
Social Support and Trust	1.1138 ^c^	(0.039)							1.091 ^b^	(0.039)
Social Participation			1.02402	(0.054)					1.014	(0.056)
Percevied Neighborhood										
Social Control					1.1647 ^b^	(0.070)			1.078	(0.072)
Percevied Neighborhood										
Social Cohesion							1.2892 ^b^	(0.113)	1.215 ^b^	(0.119)
Explanatory Variables										
Health _*t*-*1*_	6.0344 ^c^	(0.717)	6.27969 ^c^	(0.738)	6.4230 ^c^	(0.764)	6.1635 ^c^	(0.732)	6.090 ^c^	(0.736)
Married	1.1766	(0.178)	1.19200	(0.182)	1.2451	(0.191)	1.1775	(0.180)	1.199	(0.186)
Cohabitating	0.9147	(0.114)	0.94582	(0.118)	0.9337	(0.117)	0.9541	(0.119)	0.919	(0.117)
Race										
Black	0.9571	(0.143)	0.93027	(0.139)	0.9761	(0.147)	0.9670	(0.145)	1.009	(0.153)
Hispanic	0.8520	(0.142)	0.82939	(0.138)	0.8608	(0.146)	0.8496	(0.142)	0.880	(0.149)
Other	1.2224	(0.420)	1.20504	(0.418)	1.2477	(0.438)	1.2457	(0.438)	1.269	(0.444)
Poverty	0.9182	(0.117)	0.91332	(0.117)	0.9252	(0.120)	0.9116	(0.118)	0.932	(0.122)
Income										
$30,000–$59,999	1.2651	(0.195)	1.32098 ^a^	(0.203)	1.3266 ^a^	(0.207)	1.2819	(0.199)	1.265	(0.199)
$60,000 +	1.5915 ^a^	(0.400)	1.69211 ^b^	(0.425)	1.6236 ^a^	(0.413)	1.5660 ^a^	(0.396)	1.512	(0.386)
Mising Income	0.9381	(0.117)	0.93624	(0.116)	0.9415	(0.118)	0.8977	(0.112)	0.928	(0.117)
Age										
25–34	1.0431	(0.149)	1.03653	(0.147)	1.0282	(0.148)	1.0197	(0.147)	1.026	(0.149)
35–44	1.0770	(0.203)	1.04467	(0.195)	1.0232	(0.193)	1.0421	(0.196)	1.045	(0.199)
45+	1.0337	(0.413)	1.02275	(0.411)	1.0399	(0.431)	0.9935	(0.404)	1.014	(0.420)
Employed	1.3992 ^c^	(0.152)	1.41935 ^c^	(0.153)	1.4385 ^c^	(0.157)	1.4215 ^c^	(0.155)	1.417 ^c^	(0.156)
Education										
Highschool graduate	1.1369	(0.149)	1.17506	(0.154)	1.1790	(0.156)	1.1538	(0.152)	1.125	(0.150)
Some College	1.3143 ^a^	(0.201)	1.36641 ^b^	(0.207)	1.3648 ^b^	(0.210)	1.3655 ^b^	(0.209)	1.292	(0.202)
College Graduate	1.8164 ^b^	(0.537)	1.91443 ^b^	(0.562)	1.8920 ^b^	(0.555)	1.8634 ^b^	(0.544)	1.739 ^a^	(0.518)
Nonsmoker	1.4305 ^c^	(0.162)	1.42832 ^c^	(0.161)	1.4798 ^c^	(0.169)	1.4442 ^c^	(0.163)	1.465 ^c^	(0.168)
Number of children										
2	0.8174	(0.135)	0.81242	(0.135)	0.8005	(0.135)	0.8239	(0.138)	0.807	(0.136)
3	0.7767	(0.137)	0.76388	(0.135)	0.7465	(0.134)	0.7662	(0.136)	0.759	(0.136)
4 or more	0.6173 ^c^	(0.109)	0.58368 ^c^	(0.102)	0.5873 ^c^	(0.104)	0.6051 ^c^	(0.107)	0.620 ^c^	(0.111)
Constant	0.5515 ^a^	(0.174)	0.84614	(0.233)	0.4987 ^b^	(0.172)	0.4165 ^b^	(0.157)	0.258 ^c^	(0.107)
Pseudo R^2^	0.154		0.150		0.156		0.155		0.160	

The Dependent variable is good health (=1) if SRH is good, very good, or excellent; poor health otherwise. The size of the sample, n, is provided. Robust standard errors are provided in parentheses. ^a^ indicates significance at the 10 % level, ^b^ indicates significance at the 5 % level while ^c^ indicates significance at the 1 % level

**Table 3:** Marginal effects from logit estimation of social capital measures on self reported health

	Model 1		Model 2		Model 3		Model4		Model5	
Indicies of Social Capital										
Social Support and Trust	0.0088 ^b^	(0.003)							0.0099 ^a^	(0.004)
Social Participation			0.0027	(0.006)					0.0015	(0.006)
Percevied Neighborhood										
Social Control					0.0174^a^	(0.007)			0.0086	(0.008)
Percevied Neighborhood										
Social Cohesion							0.0292 ^b^	(0.010)	0.0221 ^a^	(0.011)

The marginal effects, calculated at the mean, is presented. If the variable was dichotomous the median was used as an alternative to the mean. The table also presents the standard error of the marginal effect, in the parentheses. ^a^ indicates significance at the 5% level, ^b^ indicates significance at the 1% level

In examining the contribution of socioeconomic factors in Table 2, the results suggest that as expected, the previous level of self-reported health is an important predictor of current self-reported health. The amount of financial resources available to an individual, are statistically significant in explaining the probability of reporting a favorable SRH outcome. The odds ratio on poverty is less than 1, indicating that an individual suffering economic hardship has a lower probability of reporting favorable health outcomes, though this is not statistically significant. Additionally, as income rises, the log of odds in favor of reporting favorable health outcomes increases, with the odds ratio indicating an individual in the highest income category is at least 1.51 times more likely to report a favorable level of health. Women who were employed at time (t-1) had a greater probability of reporting favorable SRH in period (t). Those who are employed are approximately 1.4 times more likely to report favorable levels of health, a result that is consistent across model specifications. While race itself was not found to be significant in the models, it is known to be highly correlated with those factors that are highly significant, including poverty, income and employment. Being a nonsmoker at time (t-1) was associated with favorable SRH at time (t), (odds ratio 1.42–1.48), while level of education attained at the birth of the child (baseline wave of the survey) was found to be positively associated with SRH. While it appears that having three or fewer children does not negatively impact SRH, having four or more children at time (t-1) lowers the probability of reporting favorable health at time (t). Race/ethnicity, marital or cohabitating status, and age were not found to be significantly associated with SRH.

As a check on the consistency of the results, a model was estimated which included all four measures of social capital (Table 2, Model 5). The results seem to reinforce the previous results, with the caveat that the social capital measure examining the behavior of the neighborhood is no longer significant. To further investigate the robustness of the relationship between social capital and SRH, an ordered logistic regression model was estimated, where SRH is based on the five categories of the original interview instrument ‘excellent’, ‘very good’, ‘good’, ‘fair’, and ‘poor’. Results support the findings of this model (results available upon request).

## Discussion

Our aim was to assess the temporal relationship between social capital and SRH in a population of vulnerable women with children. Our results suggest that aspects of social capital, notably, social support and trust, as well as perceptions of neighborhood social control and cohesion predict future SRH. Our findings are consistent with previous research into social capital and SRH over time [15–17, 19, 20]. Our findings differ from those that have found evidence of associations between social participation and mortality [21, 22]. However, it is reasonable that different aspects of social capital may predict mortality as compared to SRH, which is a measure of one’s feelings of being well or unwell. While SRH has been shown to predict mortality, the cognitive and biological processes behind this relationship remain unclear [35]. The findings also support previous research on social capital and health in underserved populations [27–29]. Furthermore, the results underscore the well-established relationship among socioeconomic status, education, and health [36–38]. Our findings reveal that trust and support seems to have components that are both individual in nature (e.g. having trusted others to help out with personal needs) and well as community-based (e.g. neighborly behaviors). This fits with previous research that has established a link between generalized social trust and SRH.

As others have discussed, social capital empowers citizens to participate in networks that also generate social capital [11]. In other words, investment in social capital is compounding. As well, it appears that social capital at the individual level is driven by the contexts of known individuals in social networks, as opposed to the contexts of random individuals [13]. Our findings support this given that the questions in the FFCWB interviews asked women about the behaviors of support and trust from persons known to the mother and the people comprising her immediate neighborhood.

These results point to the importance of the social environment as a health determinant. The findings suggest that social capital – SRH relationship in vulnerable populations is similar to that in the overall population. Such findings provide support for the idea that social capital provides a buffer effect [28]. Additionally, these findings increase the empirical support for initiatives to increase social support and neighborhood investment, and other health-in-all-policies approaches. Policy solutions targeted at increasing certain aspects of social capital, for example policies that invest in social support structures, like affordable and accessible childcare or in the development of neighborhood associations, hold promise for improving health. Given that income was also positively related to SRH, policies that focus on reducing poverty and increasing access to education and employment should be similarly promising.

A strength of this study is that it is longitudinal, approximately 3,200 women across a four-year time frame. In addition, we investigate four separate components of the complex phenomena of social capital, along with multiple social, demographic, and behavioral health determinants on SRH, thereby reducing the potential for confounding. We assess both cognitive (e.g. trust, support, and social participation) and neighborhood-related aspects of social capital. We found evidence that suggests a temporal ordering between social capital and health, though this does not infer causation. That social capital was measured at an earlier time point, and we control for health at time (t-1), we control for reverse causation. Results of both the logistic regression model and the ordered logistic regression model provide robust analyses of these relationships.

At the same time, the study has some limitations. One is that we were limited to the questions asked by the Fragile Families survey. While the measures in this study are conceptually similar to measures used in the social capital literature, the construction of measures in this study is slightly different when compared to other studies of social capital and health. Given the complexity of social capital as well as the inherent limitations of measures that are highly dependent on self-perception, it is encouraging that similar findings have been reproduced across multiple measures of social capital. At the same time, measures utilized in this study do not allow a true comparison of social capital across populations. This should be a goal of future research. That these women were located in 20 urban areas around the Unites States, another goal for future research should be to investigate the effect of context (e.g. community and policy contexts) using multilevel modeling techniques.

Another limitation of this study is that health is a very complex variable and although multiple control variables were taken into account, there is always the chance that one was missed and ultimately could skew the results. An avenue for future research is to look at dose–response relationships between various social capital constructs and health. A further limitation comes from the fact that SRH is a subjective measure, based on a complex mix between one’s interpretation of health and contextual factors. SRH, however, is an inclusive and informative measure of health status, particularly in population studies [35]. The FFCWB sample consists of low-income, urban mothers who are primarily racial and ethnic minorities, who were primarily unmarried to their partners at the time of childbirth and so findings may not extrapolate to other vulnerable populations. While our findings lend support to the idea that within this population the relationship between social capital and health is fairly similar to that in broader populations, given different measures throughout the literate more research is warranted. A final limitation is that the research offers a snapshot of how things changed over a single four-year period of time. It does not help explain how social capital changes over time or how such changes may affect health. More research is needed to explain the dynamics of social capital and its relationship with health over multiple time periods.

## Conclusion

This research adds to the literature on the relationship between social capital and health over time. Many studies are cross-sectional in nature, while this study investigates the temporal relationship between the two. The findings in this study are consistent with others’ findings in that social support and trust, and perceptions and behaviors related to the neighborhood environment from an earlier time predict health at a later time. This is true even after accounting for various social, economic, health-related, and demographic variables, as well as prior health status. In conclusion, known social contexts, as measured by social support and trust, and social participation, as well as neighborhood are important. These findings provide support to policy initiatives designed to increase income equality and access to education, as well as to specific programs that support families and neighborhoods and suggest that they would be successful at improving health over time.
